# Books Reviewed

**Published:** 1864-11

**Authors:** 


### BOOKS REVIEWED.

*A Treatise on Gonorrhœa and Syphilis, by* Silas Durkee, M. D., *consulting Surgeon of the Boston City Hospital; Fellow of the Massachusetts Medical Society; Member of the Boston Society for Medical Improvement; Honorary Member of the Medical Society of the State of New York; Fellow of the American Academy of Arts and Sciences, etc. Second edition, revised and enlarged, with eight colored illustrations. Philadelphia:* Lindsay & Blakiston, 1864.

It is now nearly five years since the first edition of the work was published; the second is now presented to the medical profession as containing the latest suggestions of the most enlightened of the present day.

The author ignores the view recently published concerning the nature of chancroid, as it has been termed, and advises all sores appearing upon the genitals under suspicious circumstances, whether they possess all the "reputed scientific attributes of a chancre or not," to be subject to caustic destruction with the view to prevent constitutional infection. He says, "It cannot do harm. If properly employed, it will occasion a small slough, after which follows a simple sore, that will heal kindly, and thus the surgeon may prevent a life of misery."

Under the head of Constitutional Treatment of Chancre, our author says, "But if the abortive plan of treatment has been seasonably executed to the extent of completely demolishing a chancrous sore, we are warranted generally speaking, in the conclusion that its previous element is also destroyed; and a resort to mercurial remedies will be uncalled for."

After quoting at considerable length the opinions of authors, and the arguments which have been presented upon both sides of the mercurial question in primary syphilis he says: "The mercurial practice has now become venerable with age, and a cloud of witnesses, both among the living and the dead, and of the highest distinction in the profession of medicine, might be adduced to testify to its efficacy; but such a marshalling of names is uncalled for." Though our author favors the use of mercury in some cases of primary syphilis, he is yet very temperate and guarded in stating his opinions upon the subject, and speaks fully upon the importance of giving it only in the smallest quantities and for the shortest periods consistent with constitutional effects—"merely to increase the redness of the gums." He acknowledges that mercury is not necessary—that all syphilitic manifestations, whether primary or secondary, may be removed without the use of mercury; but claims that in some cases accompanied by much induration, mercury materially shortens the duration of the chancrous sore. He is persuaded that secondary spmptoms occur much less frequently after the primary accidents have been treated by the use of mercury.

The work is written in clear and manly style, and for practical guide in the treatment of these diseases is safe and valuable. Many of the views entertained do not correspond with our present opinions upon the points at issue, but rather represent the views which we formerly entertained in common with the profession generally.

That portion of the work devoted to syphilitic dermatology, is especially worthy of commendation. This part is beautifully illustrated by colored plates, representing the common and more important syphilitic diseases of the skin.

*Transactions of the Medical Society of the State of New York, for the year* 1864.

We are in receipt of the volume of Transactions of the State Medical Society through the favor of the Secretary, Dr. Sylvester D. Willard of Albany, who has placed not only us but the profession generally under deep obligations for the ability and faithfulness with which he has discharged the responsible duties of Secretary, and especially for his untiring efforts to make the volume of Transactions correct and valuable. It comprises a volume of near 500 pages, which in amount, variety and value of material has perhaps not before been equalled by this society. The character and respectability of this publication must depend upon individual effort—must be sustained by individual exertion. There are a great many valuable papers contained in this volume, and we should feel under the necessity of taking them up separately for review, were it not that most of our readers will soon receive the work entire, and judge for themselves. A paper upon "*How complete is the protection of vaccination, and what are the dangers of communicating other diseases with the vaccinia,*" by A. N. Bell, M. D. of Brooklyn, is particularly worthy of mention. It received the Merit H. Cash Prize. The various questions involved are answered in direct and forcible style, and conclusions are sustained by almost conclusive proofs. This paper possesses great merit.

On the Food of Cities, by Samuel Percy, M. D. of New York, is also an attractive and important paper. "Distillery Milk Manufacture" is the topic mainly elucidated, and the statements and facts thus presented are sufficient to destroy the relish for city milk—Frank Leslie's illustrations are not necessary to complete the work.

Spinal Irritation, or Causes of Back-ache among American Women, by Charles Fayette Taylor, M. D. of New York, is also a very attractive paper. This subject is illustrated by wood cuts, while the text is philosophical, ingenious and instructive. This paper is published separately, and may be obtained from the publishers, William Wood & Co., 61 Walker Street, New York.

Regimental Surgeons of the State of New York in the War of the Rebellion 1861–4, alphabetically arranged, by Sylvester D. Willard, M. D. This is a condensed history of the Surgeons who have entered the service from New York. Name—where graduated, what service since graduation, where appointed and what changes have been made, are all arranged in tabular form. This has been carefully prepared, and will form an important item in the Medical History of this war. The profession are under deep obligations for the preparation and publication of this paper.

The volume contains many other contributions of great merit, which however we cannot notice as they deserve, and must leave the work hoping it will be carefully perused by all our readers.

Erie County Medical Society or the University of Buffalo was not represented at the last meeting of the State Society. Why this was so we are unable to explain, but we presume that the delegates chosen to represent them were detained by "severe sickness," for there can be no doubt that nothing but severe *indisposition* could have prevented the duly appointed delegates from discharging their duties.

It is most sincerely to be hoped that Erie County Society will be represented at the next meeting not by delegate only, but by worthy contribution. Buffalo College might also be set down for delegate and contribution; it is expected that such institutions shall contribute to medical science, and if they do not, the profession will draw their own inferences.

The following announcement is given for Prize Essay for 1865:

"MERIT H. CASH—PRIZE ESSAY.—This Essay for 1864 was awarded to Dr. A. Bell, of Brooklyn, and will be found in the present volume. The subject proposed for the Essay of 1865 is "*The Pathology and Treatment of Chronic Diarrhœa, contracted in Camp and Malarious Regions, illustrated by Cases.*" The subject opens a field to those who have been connected with the volunteer service of the army, and who have returned to the usual duties of private practice. Any of the volunteer surgeons of this State, now in the service, may be legitimate competitors for the prize, such being in fact residents of the State of New York.

"The prize will be about seventy dollars, or its equivalent in such form as will be most acceptable to the successful author. The Essay will also be published in the Society's Transactions."

"Competitors may send heir Essays to either of the committee, Dr. Thomas Blatchford of Troy, Dr. Edward H. Parker of Poughkeepsie, or Dr. John Ordronaux, No. 174 West 23d street, New York city, on or before the 15th day of December, 1864. In a sealed envelope should be the name of the author accompanying the Essay. The name of the successful essayist will be announced at the meeting in February, 1865.

"Albany, August 1, 1864."

*The Physician's Dose and Symptom Book, containing the doses and uses of all the principal articles of the Materia Medica and Officinal preparations; also the Tables of Weights and Measures, Rules to Proportion the Doses of Medicine, Common Abbreviations used in writing Prescriptions, Table of Poisons and Andidotes, Classification of the Materia Medica, Pharmaceutical Arrangement, Table of Symptomatology, Outlines of General Pathology and Therapeutics, by* JOSEPH H. WYTHES, A. M., M. D., *author of "The Microscopist," "Curiosities of the Microscope," etc., etc. Fourth edition. Philadelphia:* LINDSAY & BLAKISTON, 1864.

This little work is a materia medica, and a condensed theory and practice. The list of articles embraces nearly every officinal remedy and preparation, nearly every useful native medicinal plant, aud a variety of remedies recently introduced to the profession. A part is devoted to symptoms of disease and general pathology, which would perhaps be useful to the young practitioner. There are numerous tables, rules, etc., etc., the entire work compressed into the smallest possible dimensions, making it convenient as the "Pocket Companion of the Physician."

---

*A Comprehensive Medical Dictionary; containing the Pronunciation, Etymology and Signification of the terms made use of in Medicine and the kindred sciences; with an Appendix, comprising a complete list of all the more important articles of the Materia Medica, arranged according to their medicinal properties; also an explanation of the Latin terms and phrases occurring in Anatomy, Pharmacy, etc.; together with the necessary directions for writing Latin prescriptions, etc,, etc.* BY J. THOMAS, M. D., *author of the System of Pronunciation in Lippincott's Pronouncing Gazetteer of the World. Philadelphia :* J. B. LIPPINCOTT & CO., 1864.

Our limited opportunity of examining this work will for the present prevent any very extended notice, but from what we observe, we are very favorably impressed of its merits. The chief excellence, in addition to its condensed size, consists in its giving correct pronunciation of medical and scientific terms; the origin and derivation of words, and a literal translation of the various Latin phrases, sentences, etc. The work is eminently suited to the wants of the general practitioner and medical student, and considering its size, and cost, is no doubt unsurpassed in value by any similar work. We especially recommend it to the favorable notice of all medical students; it will be found invaluable.

---

*Lindsay & Blakiston's Physician's Visiting List, Diary, and Book of Engagements, for* 1865.

This annual publication has now become so extensively known and is so fully appreciated by those who have for years made it their pocket companion, that we have no occasion to speak in detail of its merits. It contains as heretofore, Almanac; Table of Signs; Marshall Hall's ready method in Asphyxia; Poisons and their Antidotes; Table for calculating the period of Utero-gestation; blank leaves for Visiting List, Monthly Memoranda, Addresses of Patients, Nurses, etc., etc. Also blank leaves for Obstetric Memoranda, Births, Deaths, etc. In its table of contents it is as full as most physicians desire for pocket use. Various sizes are made for the accommodation of all.

